# Effect of different finishing and polishing procedures on color stability of Ormocer- and methacrylate-based composites

**DOI:** 10.1186/s12903-025-07054-3

**Published:** 2025-10-30

**Authors:** Amira El-Najjar, Khaled Nour, Aya Samaha, Zainab Soliman, Farid El Askary

**Affiliations:** Operative Dentistry Department, Faculty of Dentistry, Ain Shams University, Cairo, Egypt

**Keywords:** Ormocer-based composite, Methacrylate-based composite, Color stability, Polishing techniques

## Abstract

**Background:**

The optical properties of composite restorations are influenced by composite’s surface conditions and polishing techniques, which can affect stain resistance. This study examined how surface condition and polishing techniques affect the color stability of two composite restorative materials.

**Methods:**

A total of 150 composite discs were divided into 30 experimental groups (*n* = 5) based on composite material (ormocer-based composite, Admira Fusion, and methacrylate-based composite’ Grandio), surface condition (against matrix, air, or glycerin), and polishing technique (No polishing, 1-step, 2-step, abrasive + one-step, and abrasive + two-step). Color measurements were taken using a spectrophotometer before immersion (baseline) and after 3 months of immersion in a coffee solution. Data were analyzed using ANOVA and Tukey HSD test (α = 0.05).

**Result:**

Composite type, surface condition, and polishing technique all had a significant effect on ΔE (*P* < 0.001). Admira Fusion and Grandio exhibited the lowest ΔЕ value (3.26 ± 0.15, 3.43 ± 0.08 respectively) when polished with abrasives and a 2-step technique (*P* < 0.05). Grandio showed the highest ΔЕ value (19.66 ± 0.37) when light cured in air and without exposure to any polishing technique (*P* < 0.05).

**Conclusion:**

The color stability of composites was enhanced by applying glycerin coatings prior to light curing and polishing.

## Background

Esthetic restorative materials should closely resemble natural dentition in terms of color match and stability [1]. The quality of the surface composite layer can affect its resistance to stain accumulation, particularly in the presence of the oxygen inhibition layer (OIL). This layer, which is resin-rich, is influenced by factors such as the type of monomer, initiator-activator systems, particle morphology, free radical concentration, and oxygen consumption rate [2]. Various strategies have been suggested to control or reduce this layer to enhance the surface quality of the final restoration [3, 4]. Coating composite surfaces with glycerin gel or using a Mylar strip before light curing of the composite are common techniques to prevent the formation of the OIL [5, 6]. However, the outer layer may still contain a high proportion of resin, potentially changing the optical characteristics of the cured composite [7].

According to the manufacturer’s claims Ormocers are organically modified ceramics that contain fewer free, unreacted monomers with a non-reacting C = C group [8]. Ormocer has an inorganic ceramic polysiloxane backbone that reduces polymerization shrinkage compared to dimethacrylate monomers [9]. Polymerizable side chains are also added to the polysiloxane chains, resulting in doubling the monomer conversion and enhancing the polymerization without leaving residual monomers [10]. According to Kalara et al. [11], the higher degree of polymerization of Ormocer-based composites enhances their physical properties, including color stability, compared to methacrylate-based composites. The color stability of composites is affected by both intrinsic and extrinsic factors. Intrinsic factors include the type of matrix, monomer, filler size, type, and volume, as well as photoinitiators. Extrinsic factors involve exposure to coloring beverages, especially coffee, tea, cola, and red wine, can cause varying degree of staining of composite restorations [12]. Composites can absorb liquids such as coffee and water, leading to material staining [13]. Coffee, a widely consumed beverage in daily life [14], is a common cause for yellow stains on resin composite restorations [15].

Finishing and polishing techniques are essential for removing excess material and enhancing the surface texture of final restorations. Polishing leads to a smoother surface, enhanced esthetics, and improved resistance to stain accumulation. To save time, simplified one-step polishing systems have been developed to streamline the finishing and polishing process for composite restorations. These systems are claimed to be just as effective as multi- or two-step polishing systems [16]. Nevertheless, manufacturers of the various polishing systems do not specify whether they should be used in conjunction with a pre-finishing step involving cutting or abrasive burs.

The CIELab (ΔEab) color system is a simplified system that contains all colors, including those of the light source [17]. Although the CIE 2000 (ΔЕ00) formula outperforms the ΔEab formula in assessing color difference thresholds [18], Lee et al. [19] demonstrated that the ΔEab and ΔE00 formulas could be utilized interchangeably for color evaluation.

The effect of surface conditions of composites on the color stability of composite material did not get much attention in the previous literature. Therefore, the aim of this study was to evaluate the effect of different surface conditions and various polishing techniques on the color stability of Ormocer- and methacrylate-based restorative materials. The null hypotheses were 1- type of composite material, 2- surface condition, and 3- polishing techniques will not affect the color stability of tested composites.

## Methods

Two different types of resin composite restorative materials were utilized in the current study, named a methacrylate-based resin composite (Grandio) and an Ormocer-based restorative material (Admira Fusion), both with A3 shade. A yellow-coded finishing stone and two distinct finishing and polishing systems were employed. Details of materials, including descriptions, compositions, manufacturers, and lot numbers, are provided in Table 1.

**Table 1:** Materials, description, compositions, manufacturer, and lot numbers

Material	Description	Composition	Manufacturer	Lot no.
Grandio (A3)	Light-cured nanohybrid composite.	Bis-GMA, TEDGMA, UDMA matrix. Glass–ceramic SiO2 nanofiller Filler content: 87wt%	VOCO GmbH, Cuxhaven, Germany	1,833,107
Admira Fusion (A3)	Light-cured ORMOCER composite	Ormocer monomer. Glass–ceramic, SiO2 fillers Filler content: 84 wt%	VOCO GmbH, Cuxhaven, Germany	1,905,707
Diamond abrasive	yellow coded diamond stone	Diamond coated stones(20–30 μm)	Frank, Germany	00909693
CompoSite Polisher	Two-step finishing and polishing system.	CompoSite (aluminum oxide 40 μm), CompoSite Fine (zirconium oxide 25 μm)	Shofu Inc., Kyoto, Japan	0917052
One-Gloss Set	One-step finishing and polishing system.	aluminum oxide and silicon dioxide particles.	Shofu Inc., Kyoto, Japan	0318320

### Sample size calculation

The sample size was calculated using G*Power 3.1.9.7. The effect size was calculated based on the means and standard deviations from the study by Sherif et al. [20], which compared the color stability of ormocer-based and methacrylate-based composites after immersion in a coffee solution. The t-test for the two independent variables (composites) and the power analysis was considered a priori; the calculated effect size was 0.658. With a power of 0.95 and a significance level of 95%, a sample size of 75 specimens for each composite material was deemed sufficient to detect significant differences between groups. A total of 150 discs were used with *n* = 5 discs for each experimental group.

### Study design

**Fig. 1:**
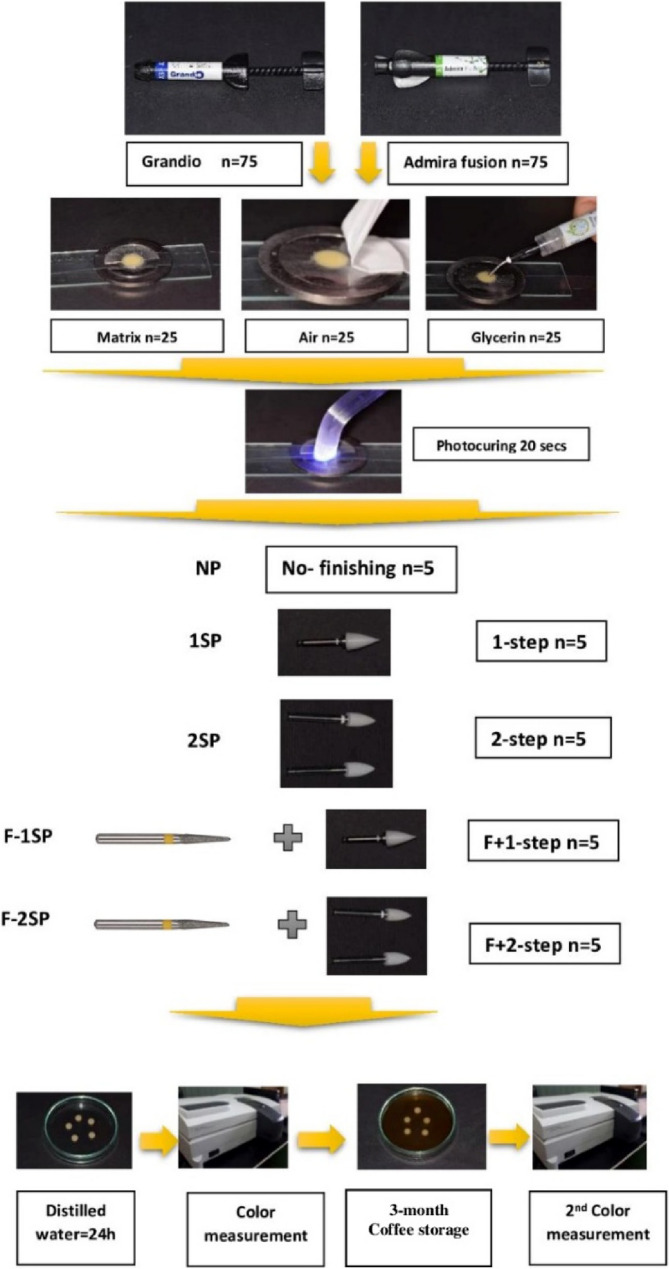
Experimental procedure flow chart

A total of 75 discs from each composite material were prepared with dimensions of 8 mm in diameter and 2 mm in thickness. The discs were randomly divided into 15 groups (*n* = 5) based on the following experimental factors: (1) material type, (2) surface condition, and (3) polishing technique. In total 150 composite discs were used, resulting in 30 experimental groups for evaluation. Figure 1 displays the design flowchart for the experimental study.

### Specimens’ preparation

All composite discs were prepared using a split stainless-steel mold with a central hole measuring 8 mm in diameter and 2 mm in thickness. The mold was placed over a glass slide covered by a Mylar strip and the material was packed into the mold using a ball burnisher and double flat instruments.

For the specimens covered with a Mylar strip before their light-curing, the composite was slightly overfilled in the mold, and the Mylar strip was placed over the material and gently pressed with a glass slide to extrude the excess material. The material was then light cured against the strip for 20 s using an LED light-curing unit (Elipar S10, 3 M ESPE). The light-curing device has a power intensity of 1200 mW/cm², which was periodically checked using a dental radiometer (Apoza Enterprise Co. Ltd., New Taipei City, Taiwan).

For the specimens light-cured in air, a Teflon tape was placed on the surface of the composite and gently pressed with a glass slide. After removing the Teflon tape, the composite was light cured for 20 s using the LED light-curing unit.

For the specimens that were covered with glycerin gel, Teflon tape was used to flatten the composite surfaces with the help of a glass slide. After removing the Teflon tape, glycerin gel (Inox gel, CERKAMED comp, Poland) was applied to the composite surface, which was light cured for 20s.

All composite surfaces that were not faced with the light-curing tip were identified with a permanent marker, and the composite discs were then prepared according to the polishing techniques proposed. Specimens from each surface condition were randomly assigned according to the polishing technique employed. Each polishing tool was used according to the manufacturer’s instructions. The polishing techniques were immediately performed after light curing of composite discs [10].

After light curing of composite discs, the discs were exposed to the following polishing techniques:

1. No-finish (control, NF): the composite surfaces were immediately stored in distilled water for 24 h at 37 °C with no polishing technique employed.
2. The 1-step polishing technique (1SP): Each composite disc was wet-polished with a rubber polishing tool using a low-speed contra-angle handpiece operated at 5000 rpm for 30 s, following the manufacturer’s instructions. The rubber tool was moved in one direction over each disc with sequential strokes until the manufacturer’s recommended time was reached.
3. The 2-step polishing technique (2SP): The finishing rubber tool was used for 15 s in one direction under water irrigation using the low-speed contra-angle handpiece according to the manufacturer’s instructions. The polishing rubber tool was then used for another 15 s under water irrigation with the low-speed handpiece. The rubber polishing tool was moved in one direction for the required time set by the manufacturer.
4. The finishing abrasive + 1-step polishing technique (F1SP): The finishing abrasive bur was moved over each disc with 10 strokes in one direction using a high-speed handpiece with water irrigation. The one-step rubber polishing tool was used as described for the one-step polishing technique.
5. The finishing abrasive + 2-step polishing technique (F2SP): The yellow-coded finishing abrasive bur was used with the same procedure described in F1SP. The 2-step polishing technique was used as described in the 2SP polishing technique.

All finishing and polishing procedures were performed by single operator who responsible for conducting the whole experiment.

### Baseline color assessment

**Fig. 2:**
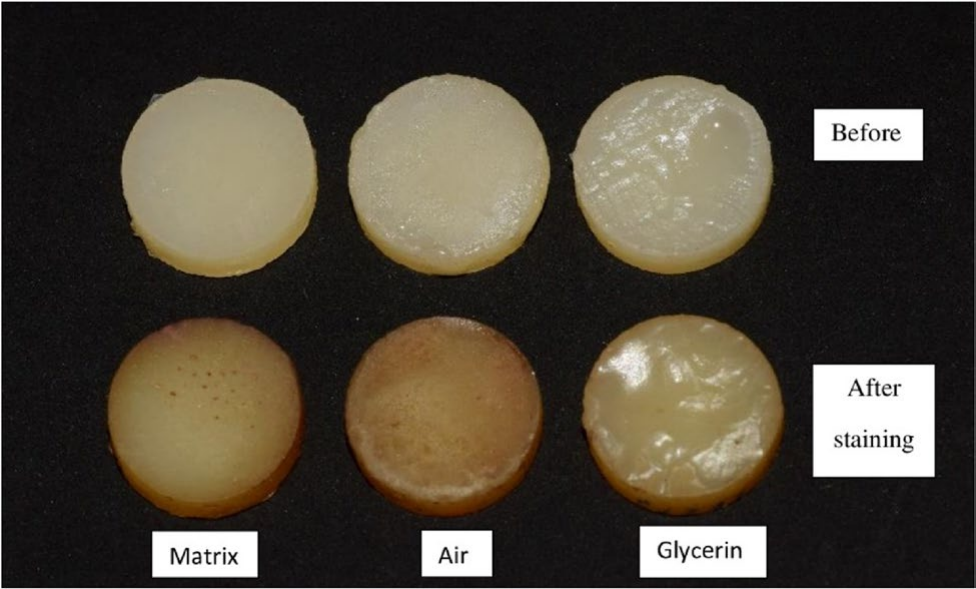
representative specimens of group NP/Grandio material in three different surface conditions (Matrix, Air, Glycerin) before/after 3-month staining procedure

The specimens were stored in distilled water at 37 °C for 24 h before baseline color measurements were taken using a Cary 5000 spectrophotometer (Agilent Technologies, USA). A baseline was recorded with the standard white polytetrafluoroethylene (PTFE) disk reference covering the reflectance port. Each disc was then mounted over the port, and the reflection of the sample surface was collected by the sphere, a baseline correction was made over the wavelength range (380–780 nm), and then the measurements were performed by pressing the bottom SCAN. Color measurement was done using the CIE L*a*b* color system. L* refers to the lightness coordinate, which has a value ranging from 0 to 100 for perfect black, and a* and b* are the chromaticity coordinates on the green-red axes (-a* = green; +a* = red) and blue-yellow axes (-b* = blue; +b* = yellow), respectively. After 3 months of coffee storage, a second color measurement was done using the spectrophotometer. The color difference was assessed before and after immersion in coffee solution according to Clarke’s formula: ΔE*ab = [(L*1- L*0)2 +(a*1-a*0)2+(b*1-b*0)2]1/2. Where L*0, a*0, and b*0 are baseline readings (before coffee immersion) and L*1, a*1, and b*1 are measurements after coffee immersion. The clinical perceptibility threshold, ΔE*ab of 3.7 units was utilized as stated by the U.S. Public Health Service (USPHS) [20, 21]. Figure 2 displays a representative photograph of Grandio composite before and after immersion in coffee solution.

### Immersion of resin composite specimens in coffee

The coffee solution was prepared by dissolving 5 g of Nescafé instant coffee (Nestlé Classic, Egypt) in 250 mL of boiling water [21]. The solution was left to cool to room temperature. Five specimens were completely immersed in 20 ml of coffee from the prepared solution and stored in an incubator at 37 °C for 3 months. A fresh coffee solution was prepared daily until the end of the storage period.

### Color assessment

After 3 months of storage in coffee solution, each disc was thoroughly rinsed with water and air-dried for 5 s. The same baseline color assessment procedures were repeated.

### Statistical analysis

Statistical analysis was conducted using SPSS 28.0 (Statistical Package for Scientific Studies, SPSS, Inc., Chicago, IL, USA) for Windows. The normal distribution of data was verified using the Kolmogorov-Smirnov test (*P* > 0.05). A 3-way ANOVA was employed to evaluate the effect of “material,” “surface condition,” “polishing technique,” and their interactions on ΔE. One-way ANOVA/Tukey’s was used for pairwise comparison. A paired t-test was used to compare the two materials. A significant level of 95% was set (α = 0.05).

## Results

The 3-Way ANOVA showed that factors “material,” “surface condition,” “finishing procedure,” and “material x surface condition x finishing procedure” had a significant effect on ΔE (*P* < 0.001).

Table 2 shows no significant ΔE difference between F1SP and F2SP in Grandio under matrix surface (*P* >0.05), with lower significant difference from other techniques (*P* < 0.05). For Admira Fusion, all polishing techniques differed significantly (*P* < 0.05). Under air surface, all techniques differed significantly in both composites, with F2SP showing the lowest significant ΔE (*P* < 0.05). Under glycerin, Grandio showed no significant ΔE difference between F1SP–F2SP and F1SP–2SP (*P* >0.05), but NF and 1SP differed significantly (*P* < 0.05). In Admira Fusion, F2SP yielded the lowest significant ΔE, with significant differences among all techniques except between F1SP and 2SP (*P* >0.05).

**Table 2:** Means ± Standard Deviations (Δ E) for the effect of materials within each finishing procedure and the effect of finishing procedure within each material in case of Matrix, Air and Glycerin on color change of the different resin composites after 3 months storage

	Matrix			Air			Glycerin		
	Grandio	Admira Fusion	*P* value	Grandio	Admira Fusion	*P* value	Grandio	Admira Fusion	*P* value
NF	8.86 ± 0.26 **D**	12.37 ± 0.07**E**	< 0.001	19.66 ± 0.37 **E**	13.90 ± 0.37 **E**	< 0.001	6.94 ± 0.22 **D**	9.21± 0.19 **D**	< 0.001
1SP	6.81 ± 0.10 **C**	8.45 ± 0.26 **D**	< 0.001	8.49 ± 0.12 **D**	8.24 ± 0.24 **D**	0.09	4.33 ± 0.17 **C**	5.18± 0.14 **C**	< 0.001
2SP	5.68 ± 0.22 **B**	7.37 ± 0.27 **C**	< 0.001	6.52 ± 0.20 **C**	6.75 ± 0.23 **C**	0.14	3.96 ± 0.13 **B**	4.17± 0.28 **B**	0.16
F1SP	4.79 ± 0.05 **A**	6.64 ± 0.15 **B**	< 0.001	5.44 ± 0.15 **B**	5.79 ± 0.20 **B**	0.02	3.68± 0.16 **AB**	3.89± 0.16 **B**	0.08
F2SP	4.54 ± 0.12 **A**	5.89 ± 0.22 **A**	< 0.001	4.67 ± 0.13 **A**	4.77 ± 0.22 **A**	0.39	3.43 ± 0.08 **A**	3.26± 0.15 **A**	0.07

Means with same capital letters within each column are not statistically significant difference at *p* = 0.05

**Fig. 3:**
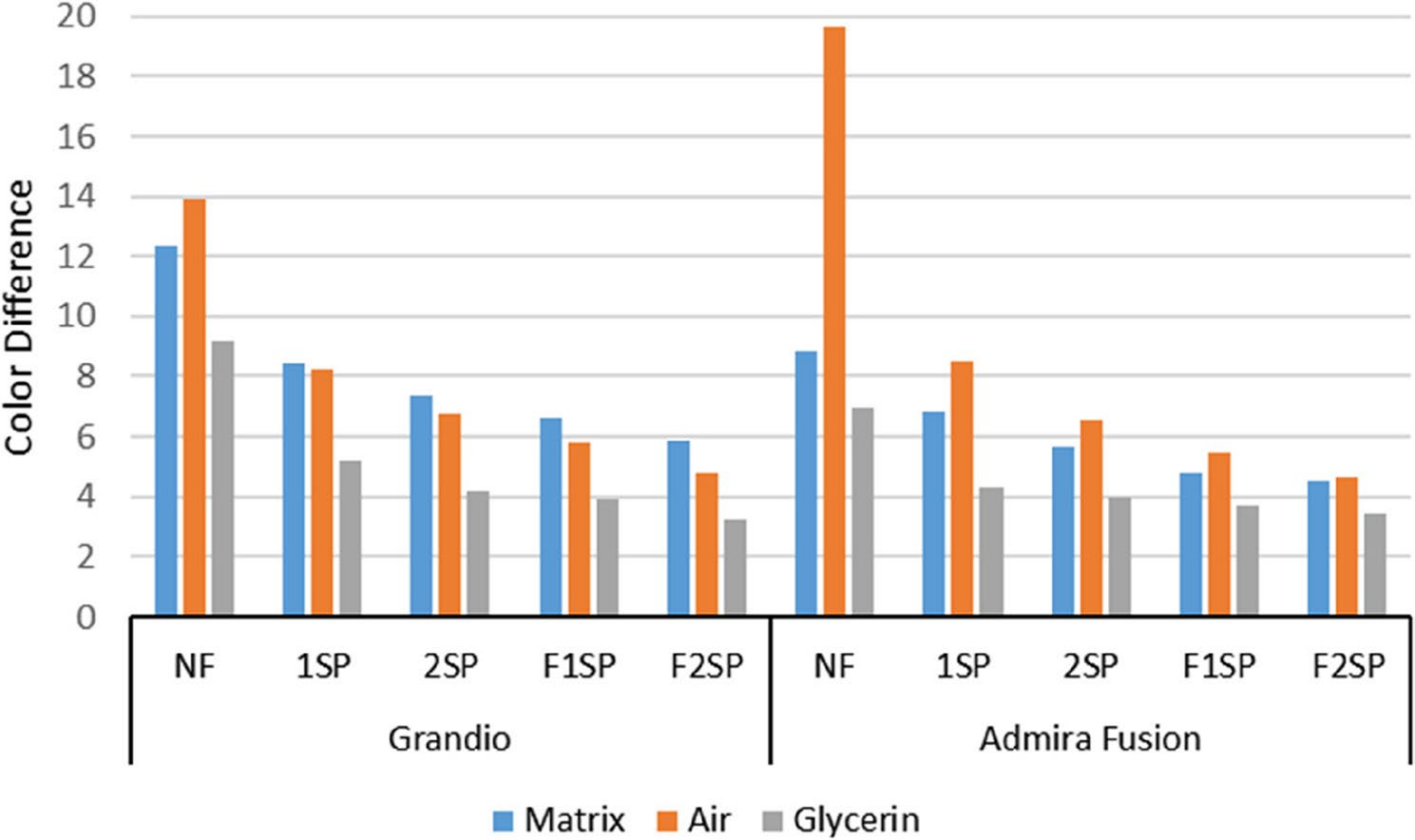
Bar graph showing the color difference (ΔE) of Grandio and Admira Fusion composite materials under different surface conditions (Matrix, Air, Glycerin) and various finishing and polishing procedures (NF, 1SP, 2SP, F1SP, F2SP)

Table 2 shows significant ΔE differences between Grandio and Admira Fusion for all polishing techniques under matrix surface (*P* < 0.05). Under air, differences were significant only in NF and F1SP (*P* < 0.05). With glycerin, only NF and 1SP showed significant differences (*P* < 0.05). Table 3 indicates significant differences among all surface conditions in both composites across all polishing techniques (*P* < 0.05). The results of ΔE for the tested materials are presented in Fig. 3.

**Table 3:** Means ± Standard Deviations (Δ E) for the effect of surface condition treatment of each finishing and polishing groups within each material on color change after 3 months storage

		Matrix	Air	Glycerin
Grandio	NF	8.86 ± 0.26 **B**	19.66 ±0.37 **C**	6.94 ± 0.22 **A**
	1SP	6.81 ± 0.10 **B**	8.49 ± 0.12 **C**	4.33 ± 0.17 **A**
	2SP	5.68 ± 0.22 **B**	6.52 ± 0.20 **C**	3.96 ± 0.13 **A**
	F1SP	4.79 ± 0.05 **B**	5.44 ± 0.15 **C**	3.68± 0.16 ** A**
	F2SP	4.54 ± 0.12 **B**	4.67 ± 0.13 ** C**	3.43 ± 0.08 **A**
Admira Fusion	NF	12.37± 0.07 **b**	13.90 ± 0.37 **c**	9.21± 0.19 **a**
	1SP	8.45 ± 0.26 **c**	8.24 ± 0.24 **b**	5.18± 0.14 **a**
	2SP	7.37 ± 0.27 **c**	6.75 ± 0.23 **b**	4.17± 0.28 **a**
	F1SP	6.64 ± 0.15 **c**	5.79 ± 0.20 **b**	3.89±0.16 **a**
	F2SP	5.89 ± 0.22 **c**	4.77 ± 0.22 **b**	3.26± 0.15 **a**

Means with same capital letters within each raw are not statistically significant difference at *p*=0.05, means with same small letters within each raw are not statistically significant difference at *p*=0.05

## Discussion

From the results of this study, the null hypothesis must be rejected. All experimental factors “material,” “surface condition,” and “polishing procedure” had significant effect on the color stability of both composite materials.

The variations in the resin composite materials’ composition could explain the differences in ΔE that could be observed between composite materials [22]. In the current study, Grandio, a methacrylate-based composite, demonstrated significantly lower ΔE values, particularly when the material was light-cured in the presence of a Mylar strip, regardless of the polishing technique employed. Also, in Glycerin group only in (NP and 1 S), Grandio material showed better color stability than Admira Fusion. This result may be attributed to Grandio’s high filler content (87 wt%), which includes silica nanofillers (20–60 nm). The filler composition of Grandio likely contributed to its superior color stability compared to Admira Fusion [23].

Llena et al. [24] found that Admira Fusion had a higher significant ΔE value than methacrylate composites following a 28-day coffee exposure period, which is consistent with the current findings. Although they employed a three-step finishing procedure, they cured the composite under a clear matrix. However, according to a study by Elmalawany et al. [25], Admira Fusion’s ΔE (5.19) was significantly lower than Grandio’s (5.57). The finishing and polishing processes were skipped even though their specimens were light cured against a matrix. The discrepancy between the results of the two research studies may be explained by the shorter staining time in Elmalawany et al. (one month) and the longer staining solution storage that occurred in the present investigation (three months). The results of Ceci et al. [26] disagreed with the results of the current study, which showed that Admira Fusion outperformed other traditional dimethacrylate resins in terms of color stability following a month of coffee staining. In Ceci et al.’s study, fine and extra-fine discs were used to polish the specimens after they had been light cured against a matrix. Since a larger filler load is linked to better color stability, this may explain the difference in the results between Ceci et al. study and the present study as all of the dimethacrylate resin composites utilized in their investigation had filler loads lower than Admira Fusion’s (84%wt.) [23].

However, Admira Fusion showed a lower ΔE value than Grandio for the Air group when no polishing was done. This might be explained by the novel matrix of ormocer material, which does not contain free dimethacrylate monomers. Ormocer is a nano-hybrid material that has 84%wt. inorganic silicate fillers and organic polymers [22]. Unlike Grandio, Admira Fusion does not contain free dimethacrylates such as Bis-GMA or TEGDMA. Grandio was predicted to form OIL after polymerization when it was light cured in air, with the unreacted monomers remaining in the material’s outer layer, whereas Admira Fusion was probably devoid of this layer. Grandio color instability may have contributed to this poorly polymerized layer [2].

For both materials, independent of the polishing procedure, the Air group recorded the highest ΔE, while the Glycerin group recorded the lowest ΔE, followed by the matrix group. The high color stability of Glycerin group may be explained by the close contact that the glycerin coat creates during the light curing process. Because glycerin could prevent oxygen from reacting with free radicals, the free radicals are able to react with monomers. The OIL’s thickness may be subsequently controlled as a result of the surface layer’s remaining monomer being reduced.

In combination with the lack of finishing processes, Bertolo et al. [4] showed that glycerin surface treatment prior to curing greatly improved the color stability of a nanohybrid composite material. Interestingly, the present investigation found that both materials produced comparable outcomes. It was reported that when glycerin was applied to the outer layer of composites and then the composite was light cured, the surface hardness was enhanced [6, 27]. Additionally, the air group had the lowest surface hardness, according to Zakiyah et al. [27]. Although more research is required, greater color stability may be the result of increased surface hardness. The use of glycerin, however, had no effect on the color stability of any of the investigated composites, according to Borges et al. [3]. This disagreed with the results of the present study, which could be explained by the variations in methodology. In the study of Borges et al., the specimens were initially cured using a mylar strip, followed by an application of glycerin, and additional light-curing process was applied.

Because of the potential for air entrapment during Mylar strip positioning, the OIL was not completely eliminated, which may have contributed to the higher ΔE value as compared to the Glycerin group [28]. However, the oxidation of unreacted monomers and photoinitiators that are not consumed after exposure to light may be the cause of the higher color changes in the Air group, particularly when no polishing techniques were used. These substances are released into an aquatic environment during aging, causing color changes [29, 30]. Three techniques were used by Marigo et al. [2] to reduce OIL: light curing using glycerin, argon gas, and Mylar strips. The outcomes were compared to groups that were cured without any barrier (air). According to the study results, the Air group had the largest ΔE value after storage in coffee, while the Mylar strip had the lowest. According to Mann et al. [31], the Air group had the highest ΔE value, the Glycerin group had an intermediate outcome, and the Matrix group had the lowest value. Due to variations in the preparation of the specimens and the materials employed, the above mentioned two investigations [2, 31] partially disagreed with the current findings, as glycerin always showed the lowest ΔE value regardless of the polishing procedure employed in this study.

The NP group showed the highest color stability; however, independent of the composite material, the polishing procedures showed the lowest color difference values. The removal of OIL that could be left behind after light curing of composites and the creation of high surface lusters could be the cause of the drop in ΔE after polishing of composite discs, leading to better stain-resistant surfaces [32, 33]. This finding was supported by Marufu et al. [34], who found that the polishing process improved the color stability of the composite. According to Bijelic-Donova et al. [35], OIL was totally removed from composite surfaces when composite specimens were finished with 1000 grit SiC papers. Additionally, Schmitt et al. [36] demonstrated that the best stain resistance nanohybrid composite surface was achieved when specimens were polished with the multi-step polishing tools.

Although previous studies showed that beverages like tea and wine significantly affect resin composite shade stability, this study was limited to a single staining solution (coffee). Storage in coffee did not fully simulate real-life conditions, presenting an additional limitation of the current study. Only two composite types were tested, despite variations in filler type, matrix composition, and content. Additionally, only color change was assessed, underscoring the need to investigate other color parameters, as well as surface hardness and roughness, in future studies.

## Conclusion

Within the limitations of our in vitro study, compared to methacrylate-based composite, the Ormocer-based composite displayed a higher color difference. Applying glycerin as a surface barrier before light curing reduced the color difference between the two composites. Polishing of composite surface, regardless of the finishing/polishing technique used, also reduced the color difference between the tested composites.

## Data Availability

Full data are available whenever requested.

## Abbreviations

OIL: oxygen inhibition layer
NF: No-finish
1SP: 1-step polishing
2SP: 2-step polishing
F1SP: finishing abrasive + one-step polishing
F2SP: finishing abrasive + two-step polishing
Bis-GMA: Bisphenol-A-Glycidyl Methacrylate
TEGDMA: Triethylene Glycol Dimethacrylate
UDMA: Urethane Dimethacrylate
